# Dressing Up for a BBQ on a Blurry Street: #TheDress Is Not Only Ambiguous in Terms of Illumination But Also in Terms of Scene Content

**DOI:** 10.1177/2041669519856037

**Published:** 2019-06-11

**Authors:** Jasna Martinovic

**Affiliations:** School of Psychology, University of Aberdeen, UK

**Keywords:** colour, ambiguity, colour constancy, illumination, #TheDress

## Abstract

Explicit or implicit assumptions about the source of illumination are a key determinant of perceived colours from the image of #TheDress. In addition, previous work showed that the extent of the processing of contextual cues in the image may be reduced in blue and black perceivers. This is a brief report of a questionnaire study which focused on the ambiguity of light direction as well as on the ambiguity of the content of #TheDress photograph itself. We replicated previous reports about the importance of perceived light direction: White and gold perceivers were more likely to report light from the back than sideways light. Descriptions of #TheDress image did not relate to perceived colour or light direction, but there were many erroneous reports and a high level of ambiguity. It is highly likely that the ambiguity of image content feeds into the importance of implicit factors that influence perceived illumination as determinants of dress colour.

Toscani, Gegenfurtner, and Doerschner (2017), Chetverikov and Ivanchei (2016), and Hesslinger and Carbon (2016) demonstrate that explicit or implicit assumptions about the source of illumination are a key determinant of perceived colours from the image of #TheDress. Hesslinger and Carbon (2016) found a slight bias towards interpreting white–blue ambiguities as blue rather than white in blue and black perceivers, hinting at the possibility of a priori differences between the two groups. Toscani et al. (2017) show that the extent of the processing of contextual cues in the image may be reduced in blue and black perceivers. Finally, individual differences in colour constancy (Witzel, O’Regan, & Hansmann-Roth, 2017) are deemed to be another important implicit factor influencing the percept.

This is a brief report of a questionnaire study which focused mainly on the reported content of the photograph itself. The hypothesis was that observers with more detailed cognitions about the content of the image and their relation to light (e.g., window letting through light or mirror reflecting light) might be more likely to perceive the dress as white and gold. The study was conducted between autumn 2015 and spring 2016. The sample consisted of 140 participants (43 men, 97 women; mean age 21 years, range 17 to 64 years) and was approved by the ethics committee of the School of Psychology, University of Aberdeen. You can find the full questionnaire and data set online (https://osf.io/a7gwk/).

Participants were first asked to look carefully at the dress and shown the image for approximately 15 seconds. Eighty-nine participants reported seeing blue and black (64%), 44 reported white and gold (31%) and 7 (5%) reported other colourations. One hundred thirty-eight participants had seen the picture before, but out of these, only 62 participants (44%) knew the real-life colour of #TheDress. This is an indicator that for a substantial number of people, viral Internet topics generate interest at a rather superficial level—and as most of our participants were first-year psychology students, it does not look favourable as an indicator of the depth of their interest in perceptual phenomena.

Participants were then asked to provide a written description of the surroundings of the dress. Nineteen percent failed to report anything (blank space or “not sure”, “don’t know”, etc.). The descriptions are summarised in Figure 1(a)—Although most of them were rather abstract (e.g., shop or room) and many of them were incorrect (e.g., blurry street), some of them did refer to light. Next, the participants were asked a series of questions about the light in the image—most importantly, where it comes from (29% front, 45% back, 24% sideways, and 3% missing data). As in Lafer-Sousa, Hermann, and Conway (2015), they also rated the warmth of the light, with somewhat cooler ratings given to the dress, dress: *M* = 2.72, *SD* = 1.13; background: *M* = 3.24, *SD* = 1.12; *t*(139) = 3.87, *p* < .001, Cohen’s *d* = 0.48. However, this did not relate to perceived dress colour, dress: *t*(131) = 0.48, *p* = .63; background: *t*(131) = 0.99, *p* = .35. Finally, blue and black and white and gold perceivers attributed the direction of light differently, χ^2^(2, *n* = 129) = 8.73, *p* = .013; Cramer’s *V* = 0.26 (Figure 1(i) to (j)). To test this further, we performed a series of Fisher’s two-sided Exact Tests while leaving out one of the three light direction responses, with no difference between front and back light (*p* = .29) and front and sideways light (*p* = .092), but significantly less sideways than back light reported by the white and gold perceivers (*p* = .004).

**Figure 1.**
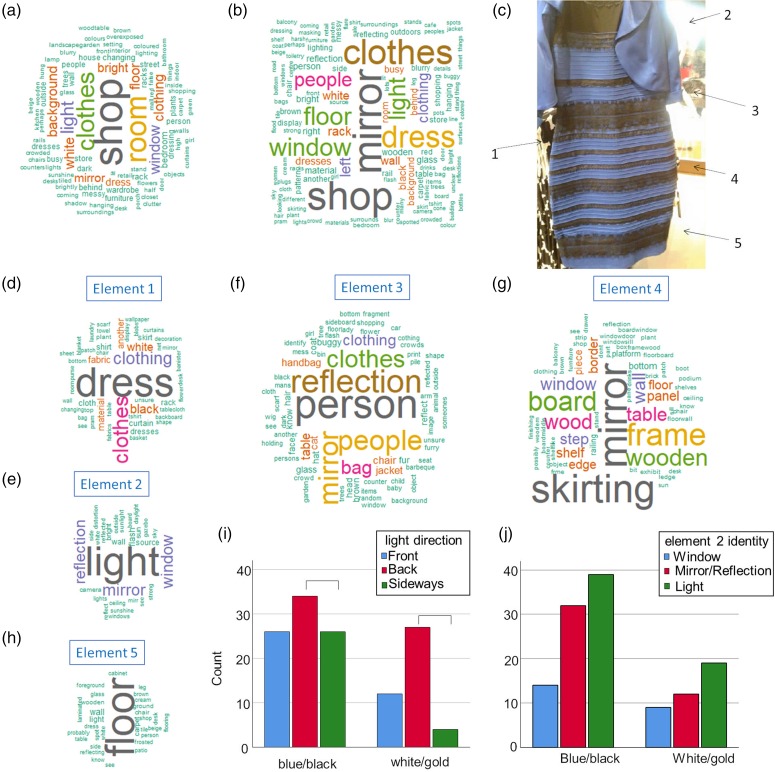
Word clouds for (a) descriptions of the image given from memory, (b) descriptions given whilst viewing the image, (c) image of #TheDress with elements whose descriptions were prompted (1–5, see sections d–h of this figure), (d–h) descriptions of various elements of the image and (i, j) bar plot of the relation between perceived colour and direction of light or description of the background.

Finally, participants were asked to describe the image of the dress while watching it and then asked to describe a series of elements of the image, each indicated by an arrow (see Figure 1(b) to (h)). The descriptions of the image given (a) from memory and (b) whilst looking were classified as correct (referring to a shop, dressing room or clothing) or incorrect (any other description). From memory, 41% of participants gave vague or incorrect descriptions of the image. This reduced to 21% when allowed to view the image while describing it. However, no link was found between dress colour perception and the accuracy of description (Fisher’s two-sided Exact Test, from memory: *p* = .46; whilst looking: *p* = .16). The importance of cognitions about background elements was also assessed, as some of them associate closely to the perceived direction of light. Descriptions (Figure 1(e)) were classified as “window” (17%), “mirror or reflectance” (33%) or just generic “light” (44%; 6% of data were missing). Again, no differences were found between blue and black and white and gold perceivers, χ^2^(2, *n* = 125) = 1.00, *p* = .61. An exploratory analysis assessed whether there was a link between background description and perceived direction of light, but again no significant differences were found, χ^2^(4, *n* = 122) = 6.49, *p* = .17.

To our knowledge, this is the first study to look at explicit cognitions about the content of the image of #TheDress. There is no noticeable link between these cognitions and judgments about illumination or perceived dress colouration—and there was sufficient power in our study to find medium-sized effects, as evidenced by the replication of the link between perceived direction of light and reported dress colouration. The number of vague or incorrect descriptions of the image was highly surprising and this is probably the main novel contribution of this report to the literature. Abstract descriptions such as “shop,” “clothes” and “room” dominate in descriptions from memory (Figure 1(a)), while in descriptions given while looking “light,” “mirror” and “window” also feature prominently (Figure 1(b)). The most ambiguous parts of the image are those elements to the top right of the dress—and they carry most information about the light. It is highly likely that the ambiguity of image content further enhances the importance of implicit factors that influence perceived illumination as the main determinants of dress colour.
